# Employing an orthotopic model to study the role of epithelial-mesenchymal transition in bladder cancer metastasis

**DOI:** 10.18632/oncotarget.11009

**Published:** 2016-08-02

**Authors:** Beat Roth, Isuru Jayaratna, Debasish Sundi, Tiewei Cheng, Jonathan Melquist, Woonyoung Choi, Sima Porten, Giovanni Nitti, Neema Navai, Matthew Wszolek, Charles Guo, Bogdan Czerniak, David McConkey, Colin Dinney

**Affiliations:** ^1^ Department of Urology, MD Anderson Cancer Center, Houston, TX, USA; ^2^ Department of Pathology, MD Anderson Cancer Center, Houston, TX, USA; ^3^ The Programs in Experimental Therapeutics and Cancer Biology, The University of Texas-Graduate School of Biomedical Sciences, Houston, TX, USA; ^4^ Department of Urology, University Hospital, Bern, Switzerland; ^5^ Department of Urology, Massachusetts General Hospital, Harvard Medical School, Boston, MA, USA

**Keywords:** SNAIL, metastasis, circulating tumor cells, orthotopic xenografts, bladder cancer

## Abstract

Epithelial-to-mesenchymal transition (EMT) has been implicated in the progression of bladder cancer. To study its contribution to bladder cancer metastasis, we established new xenograft models derived from human bladder cancer cell lines utilizing an orthotopic “recycling” technique that allowed us to isolate and examine the primary tumor and its corresponding circulating tumor cells (CTC’s) and metastatic lesions. Using whole genome mRNA expression profiling, we found that a reversible epithelial-to-mesenchymal transition (EMT) characterized by TGFβ pathway activation and SNAIL expression was associated with the accumulation of CTCs. Finally, we observed that conditional silencing of SNAIL completely blocked CTC production and regional/distant metastasis. Using this unique bladder cancer xenograft model, we conclude that metastasis is dependent on a reversible EMT mediated by SNAIL.

## INTRODUCTION

Bladder cancer (BC) poses a significant health concern to the global community [1] and once it becomes metastatic, patients have uniformly fatal outcomes. Despite this, only one advance in treatment for metastatic bladder cancer beyond cisplatin-based chemotherapy (atezolizumab [2]) has been made over the past few decades [3, 4]. The molecular mechanisms mediating BC metastasis are still poorly defined [5–8] providing the opportunity to translate new biologic insights into novel effective strategies for this disease.

Epithelial-to-mesenchymal transition (EMT) is a reversible cellular process occurring in epithelial tissues, whereby homotypic adhesion and cellular polarity are transiently lost as cells adopt mesenchymal properties and become invasive and migratory [9]. These events are driven by transforming growth factor-beta (TGFβ) and other microenvironmental stimuli that downregulate the prototypic epithelial adhesion molecule, E-cadherin, and upregulate several transcriptional repressors of E-cadherin, including ZEB1/2, SNAIL, Slug, and Twist [9]. Recent studies employing preclinical models representing squamous cell carcinoma [10] and breast cancer [5, 6, 9, 11, 12] have established that EMT is necessary for metastasis. The reverse process–“mesenchymal to epithelial transition”, or MET–may also play an important role in cancer metastasis [13]. Proliferation and invasion/migration may be mutually exclusive processes (cells do not proliferate while they are moving) [14–16], so there is possibly a growth advantage for tumor cells in the “epithelial” state. EMT has been linked to invasive BC [17–20], but its role in BC metastasis is largely unconfirmed.

In this study, we established two new murine models of metastatic BC through orthotopic recycling [21] and used a whole genome approach to characterize the changes in the expression of genes involved in EMT that occurred at critical points during metastasis. We observed that while both primary tumor cells and metastatic lesions shared epithelial markers and morphology, circulating tumor cells (CTC’s) were distinctive and displayed a mesenchymal genotype, represented by the increased expression of the transcription factor SNAIL. Furthermore, with conditional silencing of SNAIL, CTC production and regional/distant metastasis were suppressed, and then reestablished when SNAIL expression was restored. These findings support the hypothesis that transcription factors such as SNAIL promote EMT and mediate the transition to a mesenchymal phenotype in CTC’s, which is necessary for BC cells to spread to distant sites.

## RESULTS

### Recycling of mesenchymal cell lines leads to increased epithelial gene co-expression and a spontaneously metastatic xenograft model

**Figure 1:**
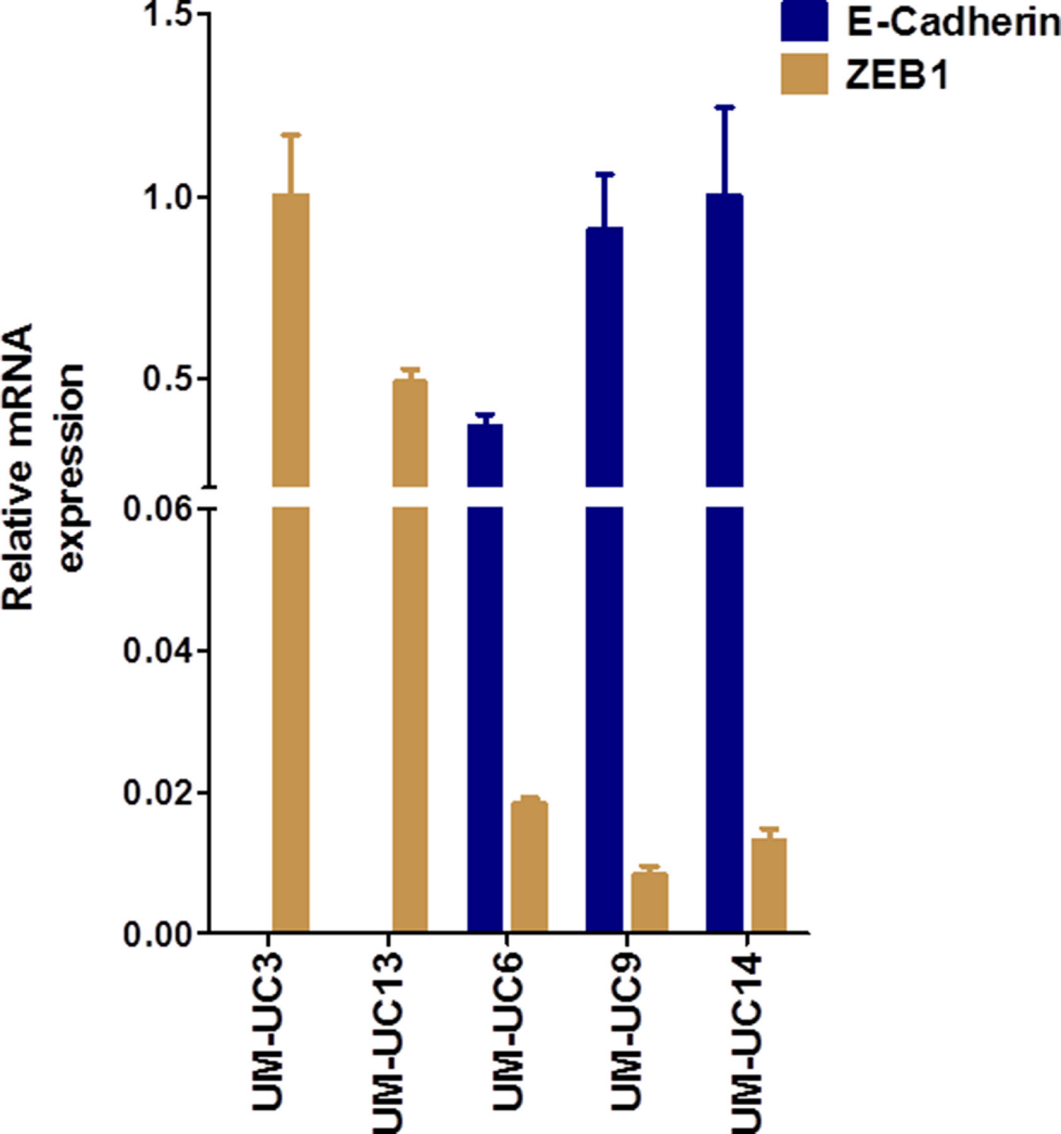
*In vivo* characterization of human bladder cancer (BC) cell lines Relative mRNA expression of labeled (luciferase and RFP) and fingerprinted cell lines grown *in vitro* prior to the first orthotopic inoculation. UM-UC3 and UM-UC13 BC cells are mesenchymal-like and showed high mRNA expression of ZEB1 and low expression of E-Cadherin compared to UM-UC6, UM-UC9, UM-UC14 BC cell lines, which are more epithelial-like.

E-cadherin and ZEB1 were expressed in a non-overlapping fashion in human BC cell lines [17], creating two distinct subgroups, “epithelial” (high E-cadherin, low ZEB1) and “mesenchymal” (low E-Cadherin, high ZEB1). Based on RT-PCR data, we classified these cell lines accordingly: UM-UC3 and UM-UC13 as “mesenchymal” and UM-UC6, UM-UC9, and UM-UC14 as “epithelial” (Figure 1). These cell lines were then stably transduced with the reporter vector (luc-RFP), sorted by FACS, and injected orthotopically into athymic nude mice.

The “epithelial” cell lines were highly tumorigenic but infrequently metastatic; lymph node (LN) metastases were only rarely identified by *in vivo* bioluminescence imaging even after recycling LN deposits (Table 1). Orthotopically implanted parental “mesenchymal” cells (UM-UC3 and UM-UC13) were initially less tumorigenic than the “epithelial” cell lines. After serial *in vivo* passaging (thereby selecting for the most proliferative tumor cells), the tumorigenicity of mesenchymal cell lines became similar to the epithelial lines. After the first orthotopic recycling, some mice developed LN and distant lung and bone metastases (Table 1). We recovered LN metastases and re-injected them into the bladder, and after 3 passages, metastases developed in most mice once primary tumors were established (Table 1). In all BC xenograft models, the latency period required for establishing tumors with a photon count of 1 × 10^10^ decreased significantly after the first cycle of orthotopic growth (Table 1).

**Table 1:** orthotopic recycling of bladder cancer cell lines

Cycle	Mice Inoculated (*n*)	Successful Bladder Grafting (*n*)	Median progression time to large tumor (10^10^photon count) (weeks)	Mice with LN Metastasis (*n*)	Mice with Distant Metastasis (*n*)
UM-UC9					
1	2	2 (100%)	14	0	0
2	5	4 (80%)	10	0	0
3	4	4 (100%)	5	1 (25%)*	0
4	16	15 (94%)	5	0	0
UM-UC6					
1	2	2 (100%)	12	0	0
2	8	8 (100%)	10	0	0
3	3	3 (100%)	4	0	0
4	3	3 (100%)	5	0	0
UM-UC14					
1	3	3 (100%)	11	0	0
2	3	3 (100%)	8	0	0
3	2	2 (100%)	3	0	0
4	16	15 (94%)	3	2 (13%)*	1 (6%)*
5	5	5 (100%)	4	0	0
6	5	5 (100%)	4	0	0
7	8	7 (88%)	3	1 (13%)*	0
8	20	20 (100%)	3	1 (5%)*	0
UM-UC3					
1	5	2 (40%)	10	0	0
2	5	3 (60%)	9	2 (40%)*	1 (20%)*
3	5	5 (100%)	6	5 (100%)*	4 (80%)*
4	5	4 (80%)	5	4 (80%)*	4 (80%)*
5	3	2 (67%)	3	2 (67%)*	2 (67%)*
6	15	13 (87%)	3	12 (80%)*	5 (33%)*
7	20	20 (100%)	3	18 (90%)*	9 (45%)*
8	18	17 (94%)	4	15 (83%)	10 (56%)
UM-UC13					
1	5	2 (40%)	16	0	0
2	5	4 (80%)	9	3 (60%)*	3 (60%)*
3	2	2 (100%)	5	2 (100%)*	1 (50%)*
4	2	2 (100%)	6	2 (100%)	2 (50%)

Labeled (luciferase and red fluorescent protein) human BC cell lines were orthotopically recycled according to previously published methodology. Growth became more aggressive with successive generations (cycles). The cell lines UM-UC3 and UM-13 are characteristically mesenchymal, and their propensity for metastasis increased with successive recycling.

*In case of lymph node or distant metastases cell suspension was performed from metastases.

### CTC counts correlate with mesenchymal primary tumor and metastatic burden

**Figure 2:**
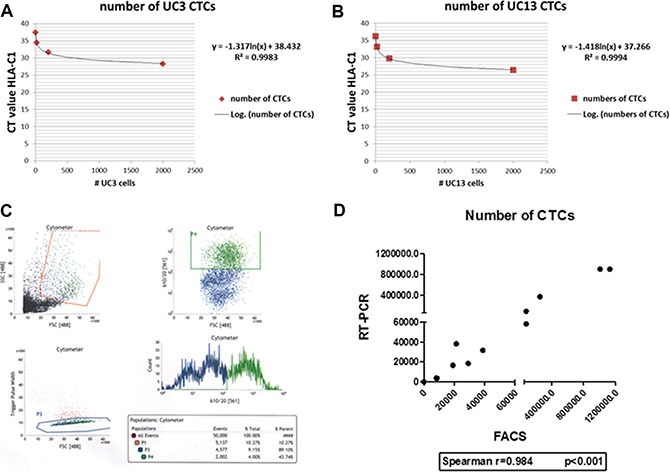
RT-PCR and FACS are effective methods of quantifying CTCs (**A–B**) Standard curves for RT-PCR quantification for UM-UC3 and UM-UC13 CTCs, respectively. (**C**) FACS plot showing identification of the CTC population (originating from UM-UC3 orthotopic tumors) in serum samples. Only the CTCs with the “strongest” signal were selected for further recycling. (**D**) Strong correlation (Spearman's rho = 0.984) of UM-UC3 CTC quantification between RT-PCR and FACS methods.

We used quantitative RT-PCR (using human HLA-C specific primers) and FACS to quantify the burden of CTCs in whole blood isolated from mice bearing recycled UM-UC3 or UM-UC13 tumors (Figure 2A–2C). There was a strong correlation between the results obtained by the two methods (Spearman *r* = 0.984, *p* < 0.001; Figure 2D), with FACS providing the advantage of enabling simultaneous isolation and purification of CTCs for further characterization.

**Figure 3:**
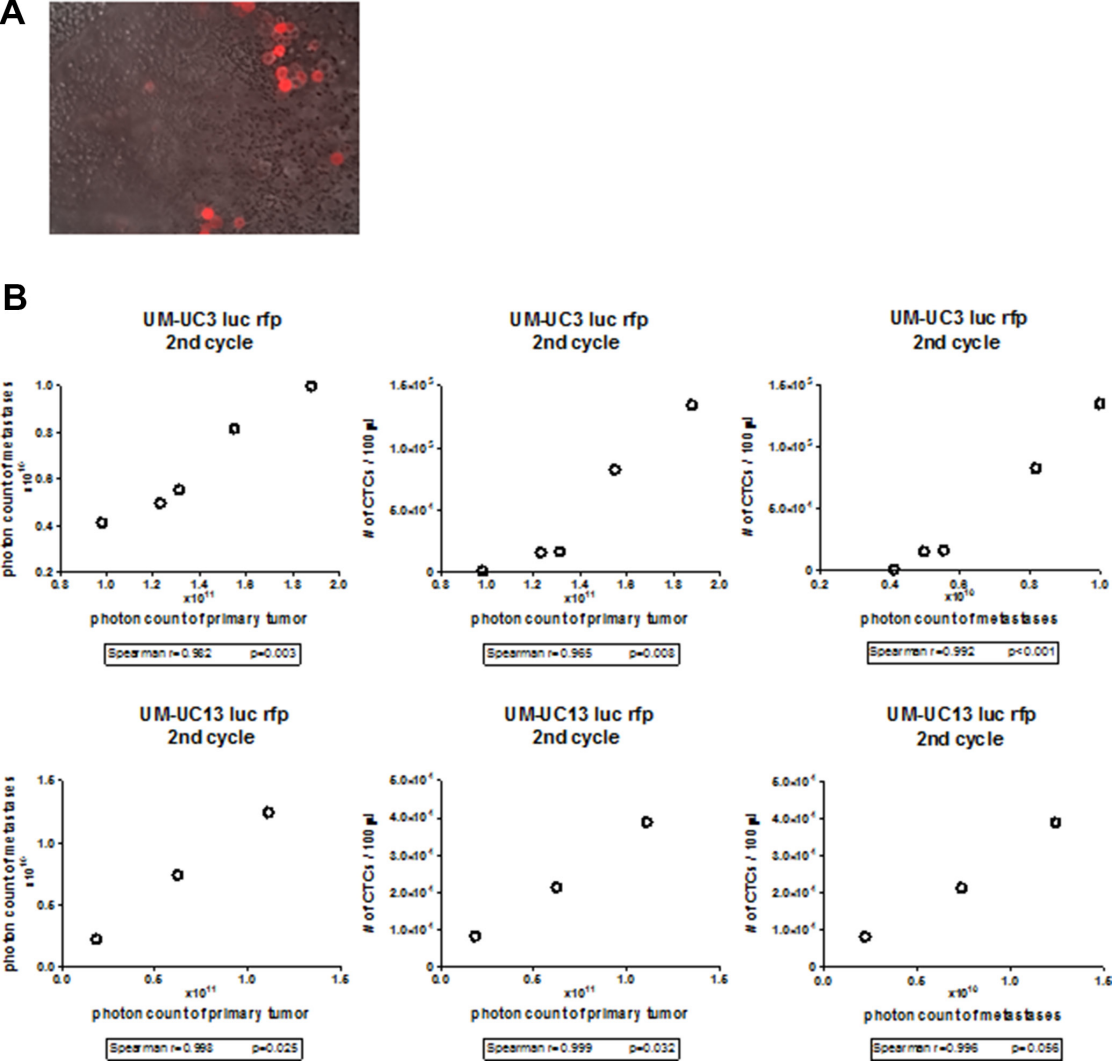
CTC correlates with primary and metastatic tumor burden in UM-UC3 and UM-UC13 xenografts (**A**) Fluorescence microscopy of blood smear originating from UM-UC3 xenografts. UM-UC3 cells are RFP-labeled. Magnification 40×. CTCs are seen as single cell and clumps. (**B**) Correlation of primary tumor size to metastasis, primary tumor size to CTCs, and metastasis to CTCs in UM-UC3 and UM-UC13 xenografts.

Xenografts from both “mesenchymal” cell lines produced large numbers of CTCs that tended to form clusters (Figure 3A) [22], and the relative burden of CTCs correlated directly with the sizes of the corresponding primary tumors and with metastatic burden (Figure 3B). Of note, no CTCs were isolated from the 3 “epithelial” human BC xenografts. Our findings establish that the presence of CTC's is associated with BC metastasis, just as they are in other solid tumors [10, 23, 24].

### CTCs have a transient mesenchymal expression profile that is lost in established metastasis

**Figure 4:**
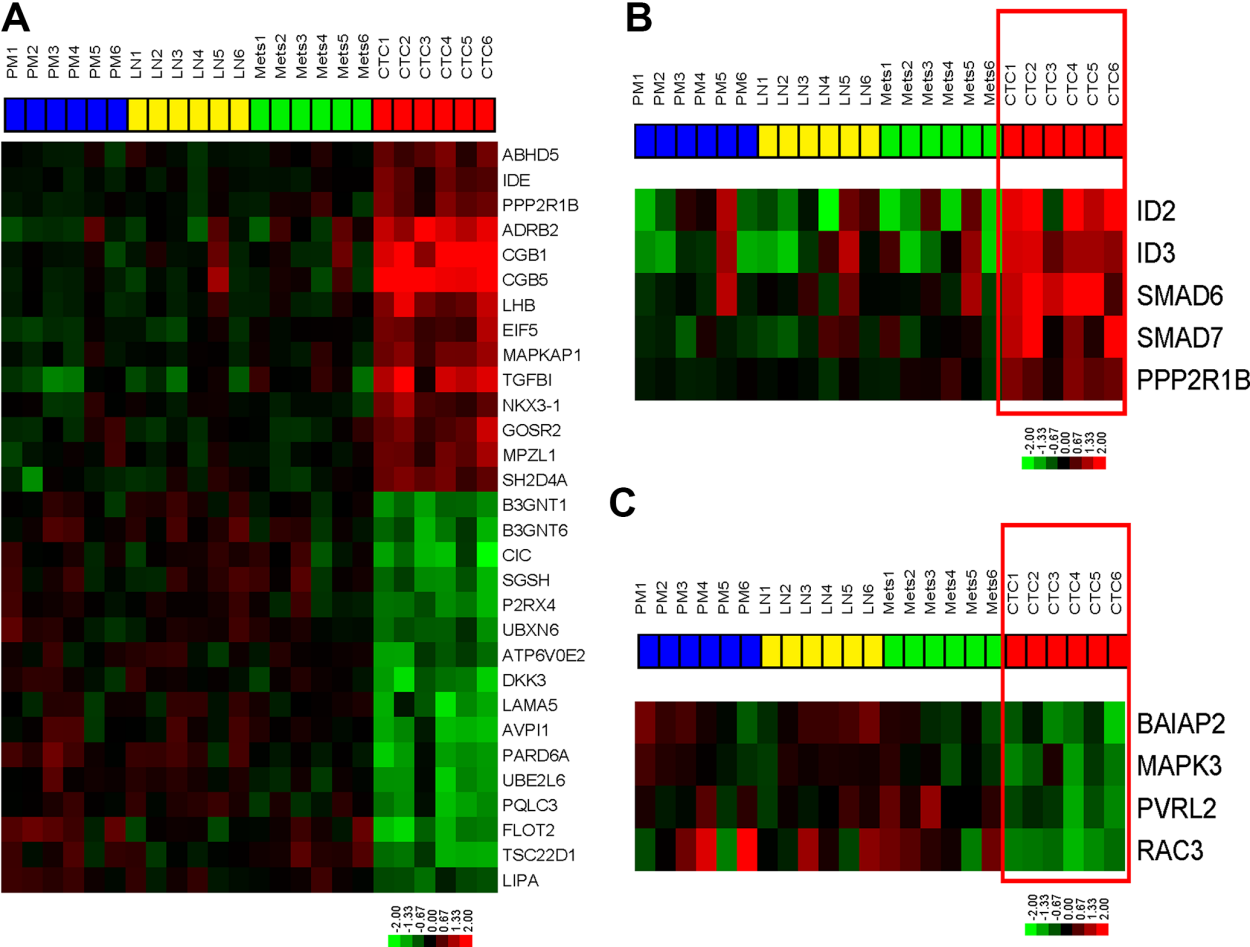
Bladder cancer CTCs are characterized by TGFβ pathway activation and loss of adherens junction gene expression Whole genome mRNA expression profiling was performed on primary tumors, CTCs, and metastases. (**A**) Heatmap of the top differentially expressed genes (FDR < 0.05, *p* < 0.001) between CTCs and others cancer-involved tissues. (**B**) Expression of TGFβ pathway targets in the KEGG pathway (*p* < 0.05). (**C**) Expression of adherens junction genes in KEGG pathway (*p* < 0.05).

We compared the whole genome mRNA expression profiles of primary bladder tumors, LN metastases, distant metastases, and CTCs isolated from mice bearing the recycled UM-UC3 xenografts. In order to understand unique aspects of CTC biology, we assessed the differentially expressed genes (FDR < 0.05, *p* < 0.001) between CTCs and other tissues (primary tumors, lymph node metastases, and distant metastases) using the class comparison tool within BRB array tools (Figure 4A, Supplementary Table S1).

**Figure 5:**
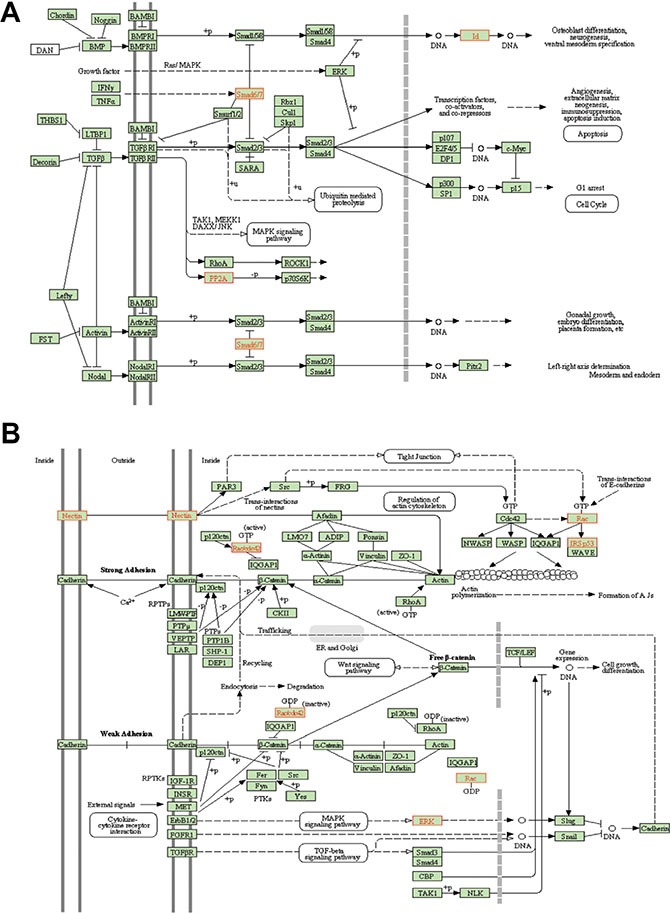
Pathway analysis of differentially expressed genes WebGestalt was used to analyze the expression profile to find patterns that matched known KEGG signaling pathways. (**A**) TGFβ pathway targets (**B**) Adherens junction genes.

WebGestalt (Figure 4B–4C, Figure 5) pathway analysis identified TGFβ and adherens KEGG pathway targets in the CTCs and the primary tumors/metastases respectively. Downstream targets of TGFβ signaling (ID2, ID3, PPP2R1B, SMAD6, SMAD7) were significantly enriched in CTCs (Figure 4B, Figure 5A), suggesting that the TGFβ pathway activation was associated with the EMT pattern observed in the CTCs [25, 26]. Conversely, adherens junction genes were down-regulated in the CTCs compared to primary tumors and metastatic deposits (Figure 4C, Figure 5B). CTCs also demonstrated low SPARCL1 expression, a feature that has been associated with metastasis in prostate cancer [27].

**Figure 6:**
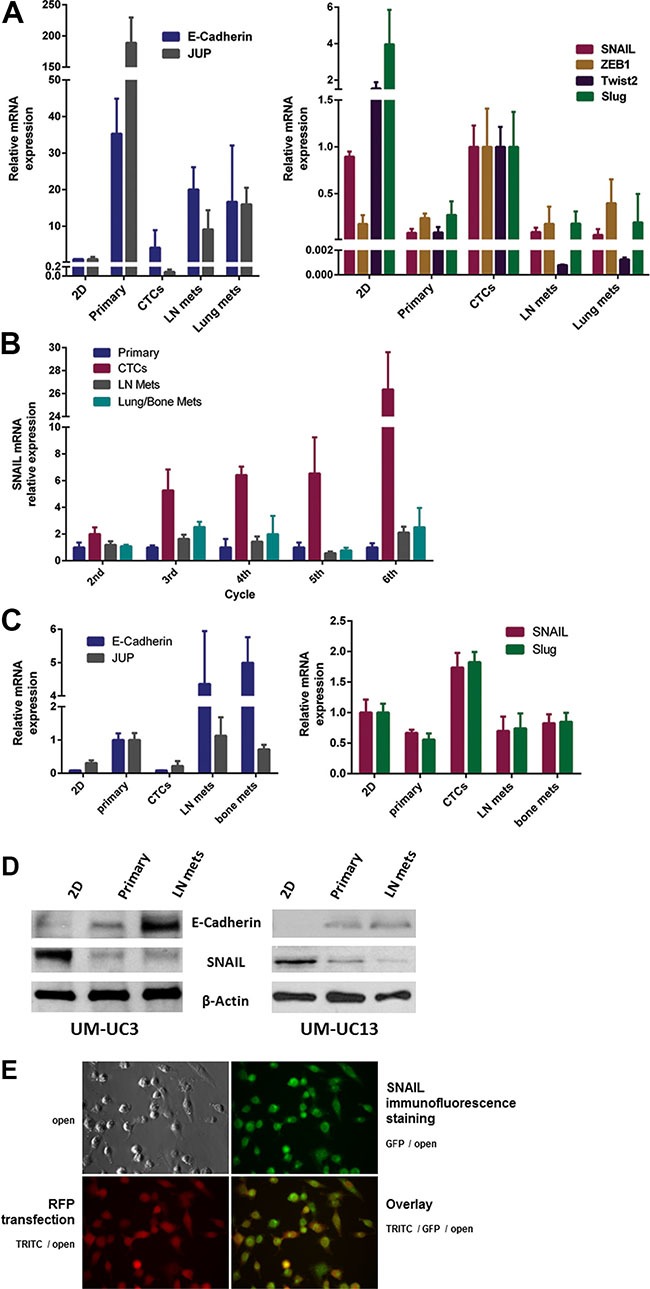
Expression of “epithelial” and “mesenchymal” markers *in vitro* and *in vivo* (**A**) Comparison of “epithelial” markers (left panel) and “mesenchymal” markers (right panel) for UM-UC3 *in vitro* (2D culture) and *in vivo* after the 3rd generation of recycling. Gene expression by the primary tumor, CTCs, lymph node (LN) metastases, and distant metastases were characterized by qRT-PCR. (**B**) Relative mRNA expression of SNAIL in UM-UC3 following successive orthotopic tumor recycling. CTCs demonstrate increasing SNAIL expression with successive generations. (**C**) Comparison of “epithelial” markers (left panel) and “mesenchymal” markers (right panel) for UM-UC13 *in vitro* (2D culture) and *in vivo* after the 3rd generation of recycling using qRT-PCR. (**D**) Immunoblots for SNAIL and E-Cadherin of 2D culture cells, primary tumors, and metastases. (**E**) Cell suspension staining (GFP) for SNAIL in CTCs originating from mice with UM-UC3 orthotopic tumors which are luc-RFP labelled.

Results of quantitative RT-PCR indicated that the CTCs were enriched for EMT biomarkers compared with the other tissue sources. Specifically, UM-UC3 CTCs expressed higher levels of mesenchymal transcription factors and lower levels of the epithelial markers E-cadherin and plakoglobin (JUP) [18] than did the primary tumors or metastases (Figure 6A). Furthermore, we confirmed that the degree of EMT in CTCs, as represented by SNAIL overexpression, was enhanced through the selection process of orthotopic recycling (Figure 6B). Using quantitative RT-PCR, we also confirmed that the CTCs originating from the second recycled “mesenchymal” cell line - UM-UC13 - expressed much higher levels of mesenchymal transcription factors SNAIL and Slug and lower levels of the epithelial markers E-cadherin and plakoglobin (JUP) (Figure 6C). RT-PCR results (SNAIL and E-Cadherin) were confirmed by Western blots (Figure 6D) and cell suspension staining of CTCs (Figure 6E).

### SNAIL mediates BC metastasis

**Figure 7:**
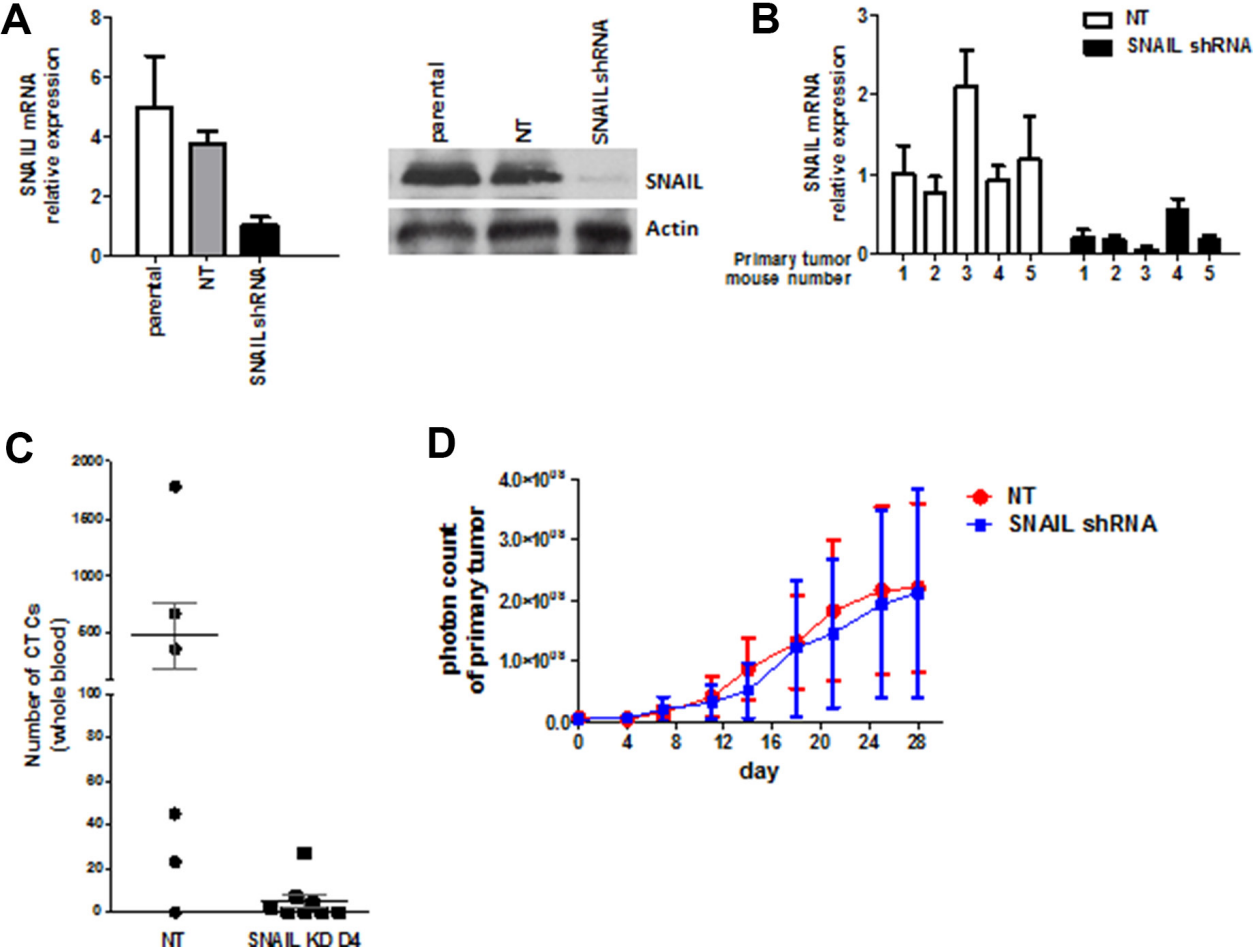
Stable SNAIL knockdown *in vitro* and *in vivo* (**A**) UM-UC3 cells were transduced with SNAIL shRNA (KD) and non-targeting control (NT). Stable knockdown was confirmed by qRT-PCR and Immunoblot analysis *in vitro*. (**B**) Confirmation of stable knockdown of SNAIL in UM-UC3 primary tumors using qRT-PCR. (**C**) The effect of stable SNAIL knockdown on CTC formation (*n* = 6–8 mice per group). (**D**) No effect of stable SNAIL knockdown on primary tumor growth could be demonstrated (*n* = 6–8 mice per group).

Whole genome mRNA expression profiling, quantitative RT-PCR, and single cell/tissue staining revealed that SNAIL (*Snai1*) seemed to play a key role in the process of EMT. Thus, we first stably transduced the recycled UM-UC3 cells with a shRNA lentiviral vector (GIPZ shRNA) and a non-targeting vector. SNAIL knockdown was confirmed *in vitro* using rtPCR and immunoblotting (Figure 7A) and *in vivo* with rtPCR (Figure 7B). Inactivation of SNAIL resulted in a significant decrease in CTCs compared to non-targeting vector (Figure 7C). There was no significant change in primary tumor growth (Figure 7D).

**Figure 8:**
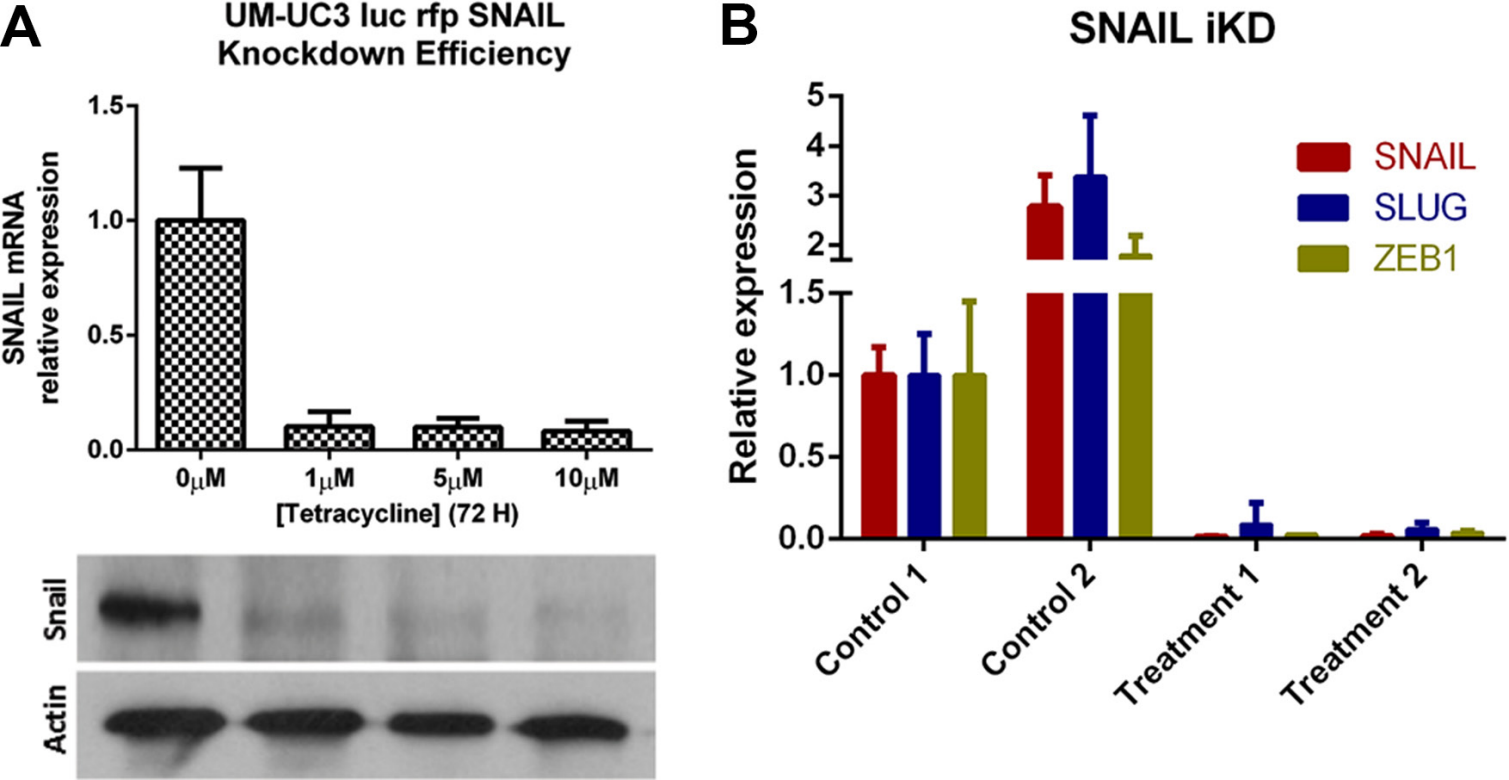
Inducible SNAIL inhibition results in other mesenchymal marker downregulation (**A**) Knockdown efficacy of the RNAi (TRIPZ shRNAmir, Tet-On^®^) transduced UM-UC3 cells (recycled from LN metastases) following puromycin selection and induction with doxycycline at different concentrations *in vitro*. qRT-PCR (top) and Immunoblot analysis (bottom). (**B**) qRT-PCR demonstrating decreased expression of SLUG and ZEB1 in primary tumors after doxycycline treatment (25 mg/kg per day); 2 primary tumors of mice treated with doxycycline (treatment 1 and 2) were compared to 2 primary tumors of control mice (drinking water only; control 1 and 2).

In order to overcome the problem of a possible off-target effect of the shRNA, we used a RNAi-based, doxycycline inducible lentiviral vector (TRIPZ shRNAmir, Tet-On^®^) targeting SNAIL to transduce recycled UM-UC3 cells (SNAIL iKD). We confirmed the efficacy of the (on/off) vector function *in vitro* with a dose response curve at the mRNA and protein level (Figure 8A). The primary tumors became more “epithelial” and less “mesenchymal” after doxycycline induced SNAIL knockdown as shown by the complementary downregulation of the “mesenchymal” markers SLUG and ZEB1 *in vivo*. (Figure 8B).

**Figure 9:**
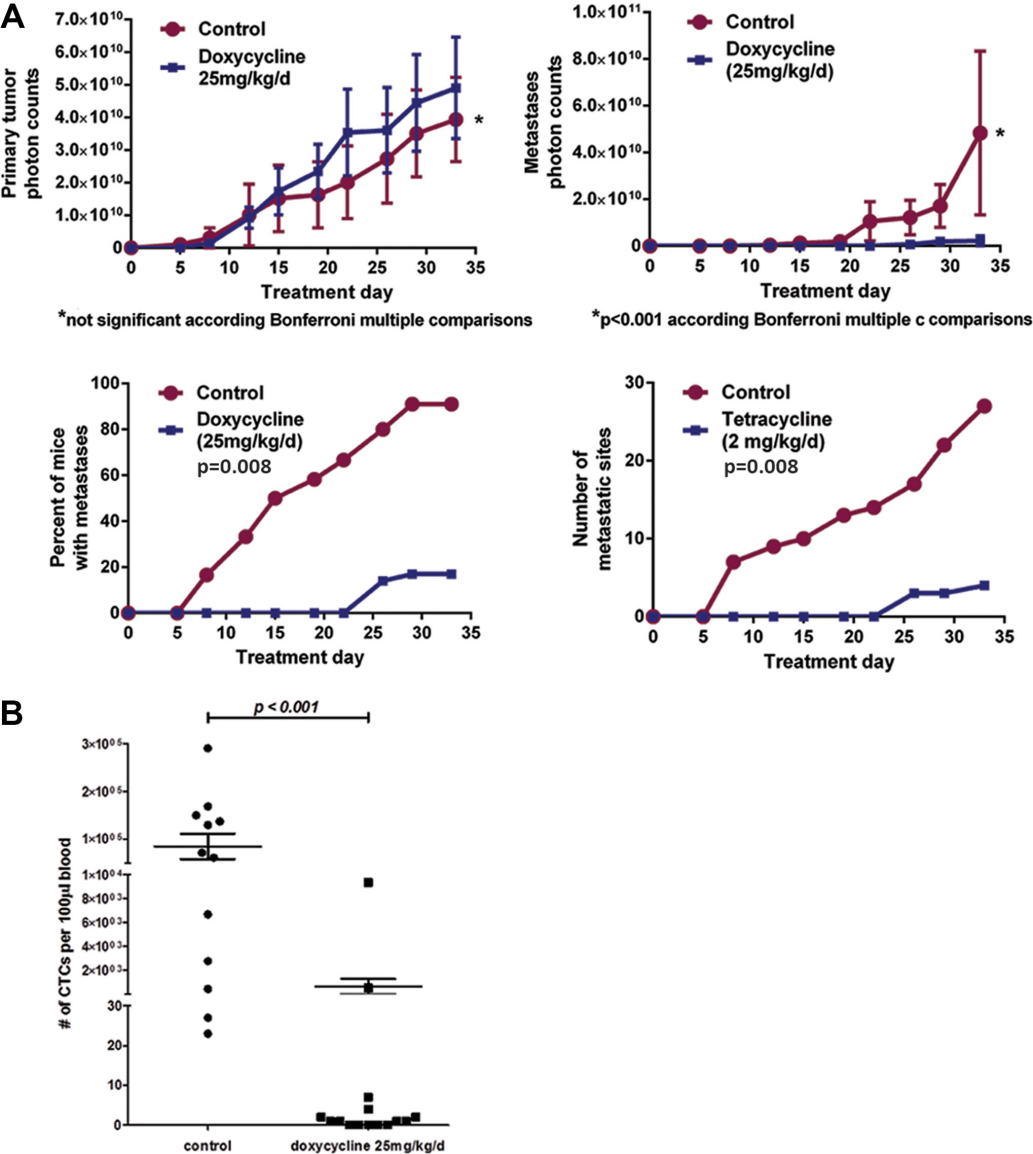
Blocking SNAIL substantially inhibited metastasis and CTC formation but not primary tumor growth (**A**) The effect of SNAIL silencing on primary tumor growth, formation of metastasis, and CTC counts at the completion of the experiment (Treatment Day 33). Control versus a doxycycline-induced SNAIL knockdown arm. (**B**) CTCs were assessed in blood collected from a terminal cardiac puncture (Mean ± SEM) and were significantly inhibited following SNAIL knockdown.

Recycled UM-UC3 cells transduced with the SNAIL iKD vector were then used for a randomized 2-arm experiment. Inactivation of SNAIL via systemic doxycycline administration resulted in a significant decrease in CTCs and metastases compared to vehicle controls but no significant change in primary tumor growth (Figure 9A–9B). To evaluate for possible off target effects of doxycycline administration on tumor metastases, we administered doxycycline in the same manner to mice with orthotopic tumors from non-transfected recycled UM- UC3 cells, and no inhibitory effect on tumor metastasis could be demonstrated. No metastases were detectable by *in vivo* bioluminescence imaging within 7 days of tumor implantation in both the UM-UC3 and UM-UC13 metastatic models (representative results for UM-UC3 SNAIL iKD in Figure 9A). Therefore, an experimental “window” appeared to exist between orthotopic tumor implantation and the establishment of spontaneous metastases that allowed for experimental manipulations directed at testing the roles of particular genetic changes in the process.

**Figure 10:**
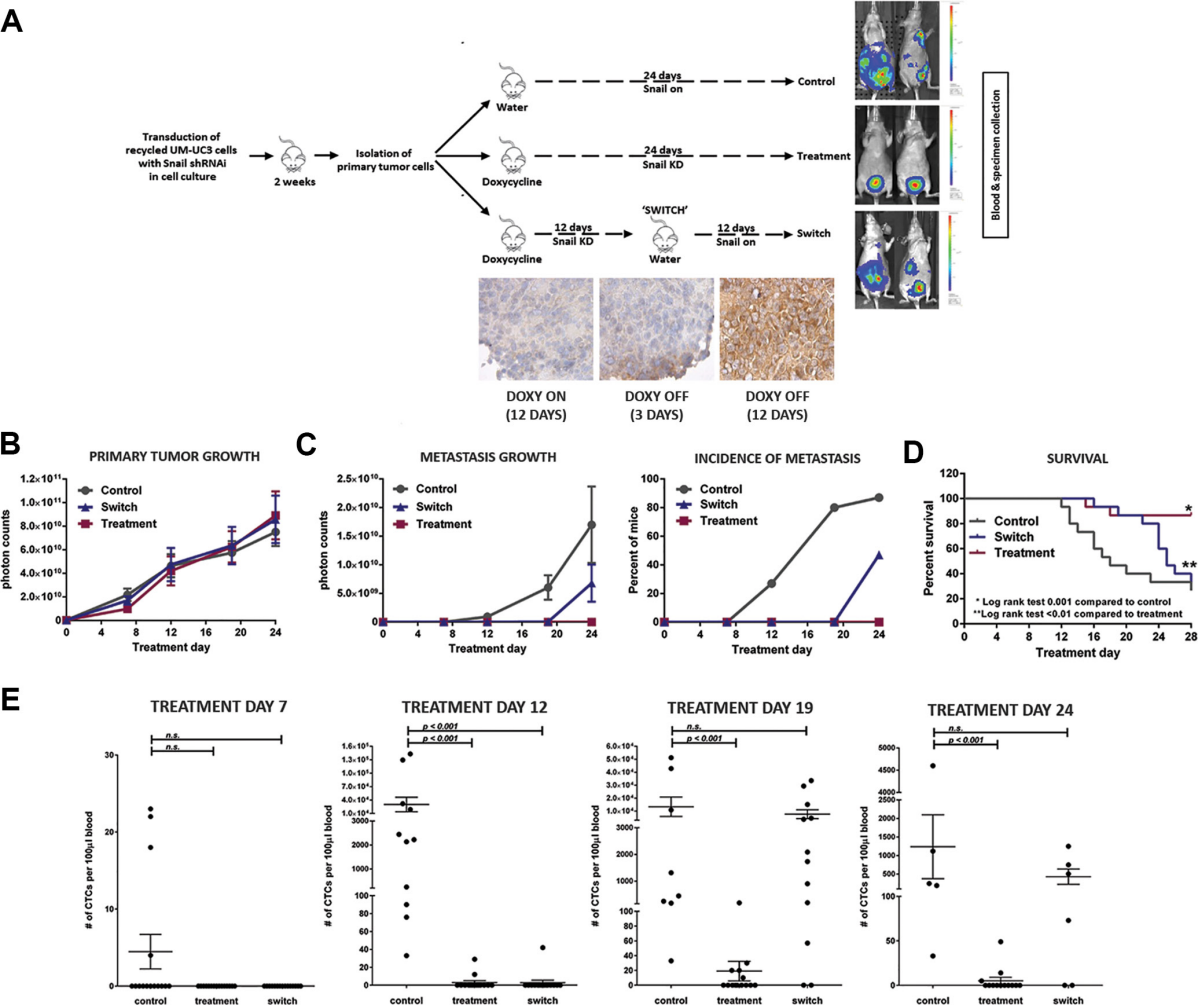
Inducible knocking down of SNAIL affects CTC and metastasis formation (**A**) Schematic of the 3-arm UM-UC3 SNAIL knockdown experiment with IHC staining for the “switch” group. After transduction *in vitro*, cells were orthotopically injected and primary tumors were grown for 2 weeks. Cells from primary tumors were isolated and inoculated in 50 mice. Mice were randomized into one of three arms (15 to 20 mice per arm). Primary tumors were established for 5 days at which point treatment commenced. The control arm received water and tumors expressed SNAIL (vehicle, no doxycycline). The mice in the treatment arm received doxycycline for 24 days continuously, knocking down SNAIL for the entirety. To assess the effect of delayed reactivation of SNAIL, mice in the “switch” group were given doxycycline until Day 12 at which point the doxycycline treatment was stopped. (**B**) Growth of primary tumors (*p* = 0.99) (**C**) Metastasis in the 3 treatment groups by photon count and percent of mice with metastasis (*p* < 0.0001). (**D**) Kaplan-Meier survival curves of the 3 different treatment groups demonstrates more favorable survival in those mice with inhibited SNAIL expression (log-rank test: *p* = 0.001). (**E**) Blood was procured for CTC quantification by tail vein aspiration (Treatment Days 7, 12, and 19) and by terminal cardiac puncture (Day 24). CTC counts were graphically compared (Mean ± SEM).

We performed a 3-arm experiment (*n* = 15 to 20 mice per arm; Figure 10A) to determine if metastasis formation regulated by SNAIL is reversible. After establishing UM-UC3 SNAIL iKD primary tumors, mice in the doxycycline and “switch” group were given doxycycline by oral gavage (25 mg/kg/d), and control animals were gavaged with vehicle only. Following treatment day 12, mice in the switch group were administered vehicle control, thereby restoring SNAIL expression by the primary tumors, seen in IHC staining (Figure 10A). We then compared the rates of primary tumor growth, tumor cell dissemination (CTCs), and the emergence of metastases. Again there were no differences in primary tumor growth among the groups (Figure 10B). None of the mice with sustained SNAIL knockdown (‘doxycycline’ group) developed metastases, whereas 87% of mice in the vehicle control group had metastases by week 4 (Figure 10C). Mice in the switch group only developed metastases after cessation of the doxycycline-induced SNAIL knockdown (Figure 10C). Consistent with their higher metastatic burden, more mice in the control group died before the planned end of the experiment at 4 weeks. Restoration of SNAIL expression in the switch group (Figure 10D) was also associated with a higher death rate in those mice. Finally, we observed the same patterns when we analyzed the CTCs, which were detectable prior to the establishment of distant metastasis. Few CTCs were present in the mice treated continuously with doxycycline, whereas mice in the control group developed significantly more CTCs, and mice in the switch group developed CTCs once doxycycline administration was stopped (Figure 10E). Collectively, these experiments clearly demonstrate that SNAIL expression is transiently induced in CTCs and is critical for spontaneous metastasis in human BC xenografts.

## DISCUSSION

Despite its relevance, there are actually very few preclinical models available for interrogating spontaneous bladder cancer metastasis. The Fidler group pioneered many of the existing models using “orthotopic recycling” [21, 28, 29], whereby human cancer cells are implanted into their native organ microenvironment promoting the transition to a more “epithelial” phenotype and enhanced primary tumor growth. Metastases are subsequently harvested and re-implanted into the orthotopic site to enrich the spontaneous metastatic phenotype. This process is repeated until essentially all mice develop metastases after a shorter latency period. Using this approach we isolated metastatic variants of the human 253J cell line that reproducibly metastasized to lymph nodes (253J B-V, recycled 5 times) or lung (253J L-IV, recycled 4 times) over 20 years ago [21]. Other groups have used intravenous inoculation of tumor cells to circumvent the need for spontaneous tumor cell extravasation [30, 31]. Intravenous injection-based experimental metastasis models, however, bypass two crucial biological steps, namely an epithelial-to-mesenchymal transition (EMT) that promotes invasion and the acquisition of circulating tumor cells (CTCs), and the reverse process (mesenchymal-to-epithelial transition, MET) at distant sites generating micrometastases [32, 33].

Early preclinical studies established that metastases arise from distinct sub-clones present within the primary tumor [7, 8, 34]. Because metastasis involves many different cell traits, it was proposed that clonal evolution selected for mutations that promoted each of the properties in sequence [35]. Our results and that of others [9–12] argue that reversible changes involving EMT (and as a consequence MET) are critically important for metastasis, challenging the notion that the metastatic phenotype is genetically hard-wired into cancer cells [21, 34], but rather that there is a central role for “plasticity” in metastasis. The recent implication of EMT in both stem cell biology and solid tumor metastasis provides one clear example of this biological plasticity [23, 36].

A comparison of the gene expression profiles between matched primary tumors, CTCs, and metastasis did not reveal dramatic differences in global gene expression among primary tumors and their metastases, but found a significantly different gene expression pattern in CTCs characterized by TGFβ pathway activation and EMT genes. Inhibiting EMT in our model system via inducible knockdown of SNAIL completely blocked CTC production and metastasis, whereas restoration of SNAIL activated both. These results complement contemporary reports that CTCs are more “mesenchymal” than matched primary tumors or metastases [22, 37, 38], and that activation of Twist-1, which similarly represses E-Cadherin, also enabled cancer cells to enter the circulation, while subsequent down-regulation of Twist1 accompanied growth at metastatic sites using a carcinogen-induced rodent model [10]. There is now accumulating evidence that EMT is promoted by tumor-host interactions [13]. Labelle et al reported that platelet derived TGFβ was sufficient to promote metastasis, while silencing TGFβ-1 inhibited the process [39]. CTCs from breast cancer patients formed aggregates with platelets and displayed evidence of TGFβ pathway activation [23], which is consistent with our observation that UM-UC3 CTC's were clustered on the blood smear and expressed downstream mediators of TGFβ signaling.

Recent evidence suggests that, in a genetically engineered pancreatic cancer and lung cancer murine models, EMT underlies chemoresistance but is not linked to metastasis [40, 41]. The different findings in these reports and our studies is unknown but may be related to different metastatic mechanisms among different tumor types, or differences in the model systems (genetically engineered conditional knockouts versus orthotopic xenografts with targeted knockdowns). Notably, even more recent evidence has linked SNAIL-mediated EMT and metastasis across multiple tumor types: hepatocellular carcinoma (SNAIL mediated EMT) [42, 43], breast cancer [44], osteosarcoma [45], and pancreatic cancer (SNAIL-mediated EMT) [46].

Since up to 40% of patients who undergo radical cystectomy with curative intent develop distant metastases [47–49], the early detection of CTCs is of utmost relevance. Past studies employing CellSearch^®^ which relies on antibodies to surface Ep-CAM to isolate CTCs yielded inconsistent results in patients with BC [50–52]. We now attribute this to our observation that Ep-CAM is restricted exclusively to human BC cells that display an “epithelial” phenotype whereas the CTCs in most preclinical metastatic models are clearly“mesenchymal”. Fortunately, great progress is being made in the advent of novel technologies to detect CTCs and associated circulating tumor DNA [53–55]. As these new platforms become commercially available, incorporating CTC metrics into routine clinical management may become possible.

Relevant preclinical models are essential to understand the mechanisms regulating metastasis. In this study we developed new orthotopic models to investigate the role of EMT in BC metastasis. Despite the limitations of orthopic xenografts [56], using this approach we demonstrated the role of SNAIL in mediating a reversible EMT in BC metastasis. These results are reminiscent of conclusions drawn from the investigation of metastasis in carcinogen-induced murine models, which highlights the importance of employing appropriate animal models to study bladder cancer. Though SNAIL is implicated in MET as a function of correlative expression data, to fully understand bladder cancer metastasis, future work employing inducible “on” systems of candidate MET regulators will be informative; as such work may generate models that generate CTCs without the ability to establish distinct metastases.

In conclusion, SNAIL-mediated EMT appears to be a requirement for human bladder cancer metastasis, particularly as cancer cells leave the primary and enter systemic circulation prior to establishing metastatic deposits. While SNAIL modulation did not alter primary tumor growth, its link to metastasis suggests a stage-specific therapeutic role for a SNAIL-targeting strategy in advanced, high-risk bladder cancer to prevent initial metastasis or progressive disease.

## MATERIALS AND METHODS

### Tissue and cell lines

Cell lines were provided by the M.D. Anderson Cancer Center Bladder SPORE Cell Line Repository and authenticated using the AmpFlSTR^®^ Identifiler^®^ Amplification (Applied Biosystems) and PCR Amplification (Applied Biosystems) kits. Cell lines were maintained as previously described [17].

### Animal protocol

Female athymic nude mice were purchased from National Cancer Institute (NCI-Frederick). The mice were housed and maintained under specific pathogen-free conditions in facilities approved by the American Association for Accreditation of Laboratory Animal Care and in accordance with current regulations and standards of the United States Department of Agriculture, United States Department of Health and Human Services. This study was carried out in strict accordance with the recommendations in the Guide for the Care and Use of Laboratory Animals of the National Institutes of Health. The protocol was approved by the University of Texas M. D. Anderson Cancer Center Animal Care and Use Committee (IACUC #110012735).

All invasive procedures were performed under inhalational anesthesia using 3% Isoflurane (Piramal Enterprises Ltd, India) in aseptic conditions. The mice were monitored daily, including weekends and holidays, with euthanasia performed using CO_2_ in the event of any of the following: tumor greater than 1.5 cm, > 20% weight loss, lethargy, inability to obtain food and/or water, labored breathing, hunched posture, abdominal distension equivalent to a pregnant mouse, or incapacitated as a result of tumor growth.

### Orthotopic recycling

Human BC cells (UM-UC3, UM-UC-6, UM-UC-9, UM-UC13, and UM-UC-14) were transduced with a lentiviral reporter vector (TRIPZ shRNAmir, Tet-On^®^) encoding luciferase (luc) and red fluorescent protein (RFP; mCherry) and adding Polybrene (Santa Cruz, TX) to increase transfection efficiency. After stable transfection of the luc-RFP reporter, cells were sorted by FACS using the Influx high-speed cell sorter (BD Biosciences). Luciferase activity was quantified by adding D-luciferin (150 μg/mL) to cell cultures and luminescence was measured using the IVIS bioluminescence system (Xenogen Co.)

To produce tumors in nude mice, sub-confluent cultures of labeled cells were harvested with trypsin, mixed with 10% FBS MEM, centrifuged at 1,200 rpm for 5 min, washed in PBS, and re-suspended in HBSS. After anesthesia induction, a low midline laparotomy incision was made and 5 × 10^5^ cells in 50 μL solution were then implanted orthotopically into the bladder wall using a 30 gauge needle. Mice were sacrificed when they became moribund, whole blood was drawn via cardiac puncture (1–1.5 ml/mouse), tumors and/or metastases were excised, minced, exposed to 1% trypsin, centrifuged (1,200 rpm for 5 min), and cultured in 10% FBS MEM. In this way, cells were recycled in female nude mice as described [17, 21] (Table 1). Mice were sacrificed when threshold tumor burden was appreciated, and tissue samples were either formalin fixed and embedded in paraffin, embedded in Tissue-Tek OCT compound (Sakura Finetek), or frozen rapidly in liquid nitrogen and stored at –80°C for RNA and protein extraction. Blood samples were put on ice and immediately processed as described below.

### *In vivo* bioluminescence imaging

Bioluminescence imaging was conducted on an IVIS 100 imaging system with Living Image software (Xenogen) as previously described [16]. In brief, animals were anesthetized before imaging in a chamber containing a 2.5% isoflurane/O_2_ mixture and injected subcutaneously with 15 mg/mL of luciferin potassium salt in PBS at a dose of 150 mg/kg body weight. 12 minutes after luciferin injection, a digital gray-scale animal image was then overlaid with a pseudocolored image representing the spatial distribution of detected photons emerging from active luciferase. Signal intensity was quantified as the sum of all detected photons within the region of interest, separately counting each primary tumor and each metastatic site.

### Immunohistochemistry

Xenograft tissue was embedded in Tissue-Tek OCT and then sections of primary tumor and metastasis were cut and blocked with 3% H_2_O_2_ in PBS, 5% horse, and 1% goat serum. We used rabbit anti-E-Cadherin (Ab40772 Abcam; 1:50) and anti-SNAIL (ab135708 Abcam; 1:50) as primary antibodies and HRP conjugated goat anti-rabbit antibody (BioRad; 1:100) for the secondary antibody. After DAB incubation, slides were counterstained with hematoxylin. Images were collected using Nikon Microphot FXA and compared to H&E stained sections processed in a conventional fashion.

### Measurement and isolation of CTCs

Blood was collected via cardiac puncture as a terminal procedure under deep anesthesia in heparin-coated collection tubes at 4°C. For further blood processing, the blood was divided into 2 aliquots, and red blood cells were lysed with 1 ml Ammonium-Chloride-Potassium (ACK) Lysing buffer (Invitrogen), and spun down to a pellet. The first pellet was lysed and total RNA was isolated for further Real-time Polymerase Chain Reaction (RT-PCR) analysis using absolute quantification to generate cycle threshold (C_T_) values for human specific HLA-C primer (Hs00740298_g1). RT-PCR analysis (in triplicate) was run together with standard isolates (0, 2, 20, 200, 2000, and 20,000 UM-UC3 cells in 100 μl mouse blood). C_T_ values of the standards were used to create a standard curve for UM- UC3 CTC, and the total number of CTC's of each whole blood sample was calculated accordingly. The other pellet was re-suspended and CTCs were counted and isolated using FACS using red fluorescent protein (RFP; mCherry) as the discriminating marker. The FACS isolated CTC pellet was finally stored at –80°C for RNA and protein extraction. We DNA fingerprinted the CTC's from both models and confirmed that the isolated cells were genetically identical to the parental UM-UC3 and UM-UC13 cells.

### Staining of CTCs and single cell suspension from tissue

Blood was obtained from tumor-bearing mice under anesthesia using cardiac puncture as a terminal procedure. Red blood cells were lysed as described, the pellet was re-suspended in 2% FBS MEM, RFP labeled tumor cells (CTCs) were isolated using FACS and transferred on 24-well plates. Cells were then washed with PBS, fixed with 4% paraformaldehyde, and stained with rabbit anti-SNAIL (GeneTex GTX100754) or anti E-Cadherin (Cell Signaling #3195; Thermo Scientific # 710161) antibodies, followed by rabbit specific Alexa Fluor^®^ 488 secondary antibody. Immunofluorescence images were collected using Olympus IX81 microscope.

### Silencing with stable (shRNA) and inducible (shRNAi) short hairpin RNA

For shRNA based knockdown, recycled UM-UC3 cells (5^th^ cycle) were plated in two 6-well plates (10^5^ cells/well) and both plates were transfected 24 hours later with the lentiviral vector of interest (GIPZ lentiviral shRNA V3LHS_328730-2; TRIPZ shRNAmir, Tet-On^®^) and a non-targeting (NT) vector, respectively. After puromycin selection (5 μg/ml), total RNA and protein lysates were collected to confirm efficacy of knockdown using quantitative RT-PCR and immunoblotting. For shRNAmir-based knockdown, shRNAi expression was induced by doxycycline (Sigma) (1–10 μg/mL) before cell lysis.

### SNAIL shRNA *in vivo* experiments

To demonstrate the effect of SNAIL expression, recycled and transduced UM-UC3 SNAIL KD cells were orthotopically injected at a concentration of 10^5^ cells in 50 μL HBBS. For the 2-arm stable knockdown versus NT vector experiment, mice (6–8 per group) were imaged after 14 and 28 days. For the 2-arm inducible SNAIL knockdown experiment, nude mice were randomized after orthotopic injection to receive either vehicle (drinking water; 250 μl/day; ‘control’ group’, *n* = 15) or doxycycline (Sigma; 25 mg/kg per day; ‘treatment’ group’,*n* = 20) by oral gavage. Mice were imaged every 3–4 days, as described in “*in vivo* bioluminescence imaging” above, for 33 days. At the end of the experiment, CTC's were collected by cardiac puncture in both experiments before mice were sacrificed, as previously described.

To demonstrate the effect of reversible SNAIL expression, recycled and transduced UM-UC3 SNAIL iKD cells were orthotopically injected at a concentration of 10^5^ cells/50 μL in 50 mice. In a 3-arm model (*n* = 15–20 for each arm), after establishing primary tumors, mice in the doxycycline and “switch” group were given doxycycline by oral gavage (25 mg/kg/d), and control animals were gavaged with vehicle only. Following treatment day 12, mice in the switch group were administered vehicle control, thereby restoring SNAIL expression by the primary tumors. We then compared the rates of primary tumor growth, tumor cell dissemination (CTCs), and the emergence of metastases. Tail vein blood (50–100 μl) was collected weekly to measure CTCs. At the end of the experiment, CTC's were collected by cardiac puncture before mice were sacrificed.

### Gene expression profiling analysis

After RNA isolation, purity and integrity were measured using the NanoDrop ND1000 spectrophotometer and the Agilent Bioanalyzer electrophoresis system, respectively. High quality RNA was then used for the synthesis of biotin-labeled cRNA using the Illumina RNA amplification kit (Ambion) as described previously [57]. Briefly, 500 ng total RNA was converted to cRNA by *in vitro* transcription, and then purified 1.5 μg cRNA was hybridized to Illumina HT12 v4 (Illumina) chip. The slides from Direct Hybridization (Illumina) were then washed and scanned with the Bead Station 500 System (Illumina). The signal intensities from the scanner were quantified using GenomeStudio Software (Illumina). Quantile normalization in linear models was used to normalize the data.

Whole genome mRNA expression profiling was performed on primary tumors, lymph nodes, metastases and CTCs. Expression data are available at NCBI GEO (GSE48496). A class comparison tool within BRB ArrayTools (National Cancer Institute, version 4) was used to select genes that are differentially expressed between the different tissue sites (6 replicates for each group). The values were averaged over replicates of samples and a two-sample *t* test was used to calculate the significance of the observations (FDR < 0.05 and *p* < 0.001). WebGestalt (http://genereg.ornl.gov/webgestalt) was used to perform KEGG pathway analyses [58]. Finally, to visualize expression patterns of specific genes of interest, expression values of these genes were adjusted to a median of zero and then analyzed with Cluster and TreeView [59].

### Real-time PCR analysis

Total RNA was isolated from xenograft tissue, FACS isolated CTCs, and 2D cultured cells using mirVANA™ miRNA Isolation Kit (Ambion, Life Technologies, CA) according to the manufacturer's protocol. Quantitative real time PCR (RT-PCR; Step One, Applied Biosystems) was used together with TaqMan^®^ Gene Expression Assays (Applied Biosystems). The comparative CT method was used to determine relative gene expression for each target gene. To normalize for the amount of amplifiable RNA, the peptidylprolyl isomerase A (cyclophilin A) gene was used as an endogenous control. All experiments were performed in triplicates.

### Immunoblotting

Cells grown in monolayer cultures were harvested at 75% to 85% confluence in lysis buffer [1% Triton X-100, 150 mmol/L NaCl, 25 mmol/L Tris, 1 mmol/L glycerol phosphate,1 mmol/L sodium orthovanadate, 1 mmol/L sodium fluoride and protease inhibitor cocktail (Sigma)] and were rotated for 30 minutes at 4°C. The lysates were centrifuged at 14,000 rpm for 10 min at 4°C to harvest supernatants. Protein concentrations were measured using the Bio-Rad Bradford protein assay (Bio-Rad Laboratories, Hercules, CA). Protein samples were boiled for 5 min in Laemmli's SDS-PAGE sample buffer and resolved on 10% SDS-PAGE gels followed by protein transfer to nitrocellulose membranes. After blocking, anti-SNAIL Ab LS-C161334 (LSBio Inc.) was used to investigate the SNAIL knockdown. Species specific (Anti-Rabbit) secondary antibody was used for probing, and the enhanced chemiluminescence system (Amersham Biosciences, Piscataway, NJ) for detection.

### Statistics

Statistical analysis was performed using GraphPad Prism Software (GraphPad, San Diego, CA). As appropriate, raw data or percentages were compared by unpaired Student's *t*-test or Mann Whitney test. Tumor growth curves in xenografts were analyzed using Two-Way ANOVA with Bonferroni multiple comparisons. Spearman's rank correlation coefficient was used to determine the significance of correlations between two continuous variables in a non-parametric fashion. Statistical significance was set at *p* < 0.05.

## SUPPLEMENTARY MATERIALS TABLE




