# Supplementary material for: Employing an orthotopic model to study the role of epithelial-mesenchymal transition in bladder cancer metastasis
Source: Oncotarget. 2016 Aug 2;8(21):34205–22. doi: 10.18632/oncotarget.11009 (PMC5470961; doi:10.18632/oncotarget.11009)
Supplement: Supplementary file 1 [file oncotarget-08-34205-s001.pdf]

# Employing an orthotopic model to study the role of epithelial-mesenchymal transition in bladder cancer metastasis

## Supplementary Materials

**Supplementary Table S1: Differentially expressed genes in CTCs versus the other tumor sites with FDR< 0.05, $p < 0.001$, and fold-change ≥ 1.5 using the class comparison tool within BRB array tools.** See Supplementary_Table_S1
